# Auditory Dysfunction in Patients with Cerebrovascular Disease

**DOI:** 10.1155/2014/261824

**Published:** 2014-10-23

**Authors:** Sadaharu Tabuchi

**Affiliations:** Department of Neurosurgery, Tottori Prefectural Central Hospital, 730 Ezu, Tottori, Tottori 680-0901, Japan

## Abstract

Auditory dysfunction is a common clinical symptom that can induce profound effects on the quality of life of those affected. Cerebrovascular disease (CVD) is the most prevalent neurological disorder today, but it has generally been considered a rare cause of auditory dysfunction. However, a substantial proportion of patients with stroke might have auditory dysfunction that has been underestimated due to difficulties with evaluation. The present study reviews relationships between auditory dysfunction and types of CVD including cerebral infarction, intracerebral hemorrhage, subarachnoid hemorrhage, cerebrovascular malformation, moyamoya disease, and superficial siderosis. Recent advances in the etiology, anatomy, and strategies to diagnose and treat these conditions are described. The numbers of patients with CVD accompanied by auditory dysfunction will increase as the population ages. Cerebrovascular diseases often include the auditory system, resulting in various types of auditory dysfunctions, such as unilateral or bilateral deafness, cortical deafness, pure word deafness, auditory agnosia, and auditory hallucinations, some of which are subtle and can only be detected by precise psychoacoustic and electrophysiological testing. The contribution of CVD to auditory dysfunction needs to be understood because CVD can be fatal if overlooked.

## 1. Introduction

Auditory function is complex, as it anatomically necessitates the transmission of an auditory signal from the ear to the auditory cortex and further processing to facilitate the perception and recognition of sound. Hearing disturbances profoundly affect quality of life and can occasionally be a cause of life-threatening disorders of the central nervous system. Auditory dysfunction is usually masked by other neurological issues and it can be difficult to evaluate in patients with cerebral stroke. Cerebrovascular disease (CVD) is the most prevalent neurological disorder and it can cause various types of auditory dysfunction. This study reviews current understanding of interactions between CVD and auditory dysfunction.

## 2. Review

### 2.1. Cerebral Infarction and Cerebral Ischemia

#### 2.1.1. Bitemporal Infarction and Ischemia

The clinical syndrome of cortical deafness with bitemporal infarction has become established since it was first described in 1883 [1]. Cortical auditory disorders considerably vary and the variety of descriptions, such as auditory agnosia [2], apperceptive agnosia [3], pure word deafness [4], central deafness [5], and reversible cortical (central) auditory dysfunction [6, 7], indicates substantial overlap and thus a spectrum of related auditory processing disorders. The central auditory nervous system consists of cortical, subcortical, and interhemispheric connections [8]. Differences between syndromes might depend on the degree to which the primary cortical processing, accessory, and efferent auditory systems are involved [9].

#### 2.1.2. Anterior Inferior Cerebellar Artery (AICA) Infarction

An acute ischemic stroke in the distribution of the AICA is associated with facial weakness, hypalgesia, ataxia, vertigo, hearing loss, and nystagmus. Some patients lack or may not be aware of the symptom associated with brainstem or cerebellar signs. Among them,sudden hearing loss is a common sign of an AICA infarct [10–12]. The major responsible focus is the cochlear injury, which results in sudden unilateral deafness and is frequently associated with tinnitus. Cerebellar infarction is relatively infrequent and comprises 2.3% of all patients with acute stroke involving the posterior inferior cerebellar artery (PICA; 49%) and AICA (20%) regions [13]. Magnetic resonance imaging (MRI) in one study found that the most common site affected by an AICA infarction was the middle cerebellar peduncle [10]. A sensorineural hearing loss was found in 92% of AICA infarctions in that series. The most common mechanism of AICA infarction is an atheroma or thrombus in the parent basilar artery that blocks the AICA [14]. An auditory disturbance has also been identified as a prodrome of AICA infarction [15, 16]. The AICA supplies the dorsolateral pons, middle cerebellar peduncle, inner ear, vestibulocochlear nerve, and anterior inferior cerebellum including the flocculus [17, 18]. Because the blood supply to the peripheral auditory system arises from the internal auditory artery that is ordinarily a branch of the AICA, partial ischemia in the AICA territory could lead to an isolated acute auditory syndrome such as hearing loss and tinnitus [15]. Sudden deafness with an AICA infarction is usually due to a dysfunction of the cochlea resulting from ischemia to the inner ear [10]. Because symptoms and signs of cerebellar infarcts are very similar to benign peripheral labyrinthine disorders, many patients with cerebellar infarcts are likely to be overlooked unless they are assessed by CT/MRI [13]. The possibility of AICA infarction particularly in elderly patients with sudden deafness and risk factors for CVD should be considered, even if classic brainstem or cerebellar signs are absent.

#### 2.1.3. Non-AICA Origin Posterior Circulation Infarction

Huang et al. described seven patients with sudden bilateral hearing loss caused by vertebrobasilar occlusive disease [19]. Toyoda et al. described two patients with basilar artery occlusion in whom bilateral hearing loss warned of impending stroke [20]. Sudden deafness due to right vertebral artery dissection has also caused vertebrobasilar ischemic stroke [21]. Sudden deafness due to non-AICA territory infarction is mostly associated with an infarct in the territory of the PICA [22]. The labyrinthine artery (auditory artery and internal auditory artery) usually originates from the AICA, but it can occasionally originate from the PICA or directly from the basilar artery [23]. Non-AICA territory posterior circulation infarcts result in unilateral and/or bilateral acute hearing loss. We should take this pathology into consideration especially in case of bilateral acute hearing loss.

#### 2.1.4. Cerebral Venous Thrombosis (CVT)

There are several reported cases of CVT affecting the eighth cranial nerve. CVT presenting as multiple lower cranial nerve palsies including eighth nerve was reported [24]. Kim et al. reported CVT mimicking acute unilateral vestibulopathy [25]. Assessment by MRI identified extensive CVT involving the superior longitudinal sinus, the straight sinus, and the proximal portion of both transverse sinuses in a patient with CVT sustained reversible bilateral sensorineural hearing loss [26]. Crassard et al. described a patient with lateral sinus thrombosis who presented with acute hearing loss without vertigo or imbalance. Cochlear venous blood collected by the cochlear vein drains through the labyrinthine vein into the inferior petrosal sinus or directly into the transverse sinus. Thrombosis of the transverse sinus might increase cochlear pressure and induce anoxic changes as a result of impaired drainage or thrombosis extending to the cochlear or labyrinthine veins [27].

### 2.2. Intracerebral Hemorrhage (ICH)

#### 2.2.1. Bilateral ICH

Cortical deafness is a rare condition that occurs with bilateral temporal lobe lesions [1] or with bilateral subcortical lesions interrupting the ascending auditory pathways [28]. In cortical deafness, patients appear deaf, although some reflex responses, such as turning towards a sudden loud sound, may be preserved. With time, some auditory capacities may reemerge. Other patients remain permanently deaf [8].

Cortical deafness can follow bilateral hypertensive putaminal hemorrhage [29–31]. Bilateral hypertensive putaminal hemorrhage caused cortical deafness in two patients, possibly due to complete transaction of the acoustic radiation and cell degeneration in the medial geniculate body with putaminal hemorrhage on both sides and an auditory system that might have been dominant in the contralateral hemisphere [29]. Bilateral damage due to acoustic radiation of the temporal lobe without involving the medial geniculate body might have caused the cortical deafness in a patient described by Nishioka et al. [30].

Auditory agnosia refers to impaired perception restricted to certain classes of sounds. Word deafness, the most striking type of auditory agnosia, is the incapacity to recognize speech sounds [8]. Pure word deafness, inability to understand spoken words, despite intact hearing, speech production, and an ability of reading, is rare [4]. Word deafness mostly occurs as a result of bilateral temporal lesions, interrupting the connections between the two primary auditory cortices to Wernicke's area. Pure word deafness is produced by a posterior unilateral temporal lesion [8]. In contrast to sensory aphasia, reading and writing are preserved in word deafness because they are not fed by auditory input.

#### 2.2.2. Cerebellar Hemorrhage

Acute vestibular syndrome due to cerebellar hemorrhage is similar to those of acute cerebellur infarction. Surgical evacuation of a subpial hematoma partially improved hearing loss and tinnitus that comprised the initial symptoms of a cerebellar hemorrhage [32]. Crossed pontocerebellar fibers as well as the cochlear and vestibular nerves might have been damaged by hemorrhage of the middle cerebellar peduncle. The right acoustic nerve was remarkably swollen by the hematoma [32].

#### 2.2.3. Brain Stem Hemorrhage

Central pontine hemorrhage was associated with auditory dysfunction in four patients [33]. Damage to the medial superior olivary nuclei and to the trapezoid body involving both afferent and efferent fibers was responsible for the symptoms. Bilateral total deafness due to a single pontine hemorrhage involved an inactivated trapezoid body in a patient [34] whose other pontine auditory structures were spared. All of the above patients were conservatively treated with medication and the symptoms were reversible. The auditory symptoms associated with brain stem stroke include hearing loss, phantom auditory perceptions (auditory hallucinations), and hyperacusis [8]. Smaller brain stem hemorrhage involving the caudal pons can cause hearing impairment and hallucinations that can be either unilateral or bilateral [35]. Hyperacusis is the least common of the auditory complaints; it has been reported for a patient with a bilateral tectal midbrain hemorrhage [36]. Peduncular hallucinations can occur with midbrain strokes, but they are predominantly visual with an occasional minor auditory component, such as seeing people who are “whispering” [37]. Sometimes central auditory disorders in stroke are initially mistaken for acute psychosis [8].

Bilateral ICH, cerebellar, and brain stem hemorrhage are relatively frequent in elderly patients, and the degree and type of auditory dysfunction might considerably vary from a complete bilateral hearing loss to a partial or unilateral hearing disturbance and even auditory hallucinations. Thus, the incidence of auditory dysfunction due to bilateral ICH, cerebellar, and brain stem hemorrhage might be underestimated or misdiagnosed as presbycusis. Symptoms can be subtle and/or transient and thus difficult to diagnose. Thus, auditory function should be carefully assessed in elderly patients during the acute and chronic phases.

### 2.3. Subarachnoid Hemorrhage (SAH)

#### 2.3.1. Cerebral Aneurysm

Distal AICA aneurysms can cause auditory disturbances and cause SAH and complete ipsilateral deafness. The reported incidence of aneurysms of AICA is about 0.1% of all cerebral aneurysms; only 56 patients with such aneurysms have been described in the literature [38, 39]. Among them, intracanalicular (internal auditory artery) aneurysms are extremely rare, as only six patients have been documented [40–45] and all of them were deaf. Most distal AICA aneurysms present as SAH (82.5%) and less frequently as only cerebellopontine mass signs (17.5%) [39]. The cause of acoustic nerve palsy can be ischemic [46, 47], direct nerve compression [46], or the presence of hemosiderin after SAH within the inner ear [48]. If the internal auditory artery aneurysm compresses the auditory nerve, the aneurismal sac should be removed to achieve good recovery of nerve function [38]. The technical difficulty of neck clipping in this site may be attributed to its location and to the adherence of the aneurismal neck to the surrounding structures [39]. Castaigne et al. were the first to successfully treat an intrameatal aneurysm surgically in 1967 [40], and Hitselberger and Gardner presented another case the following year [49] and then Hori et al. surgically trapped an intrameatal aneurysm [42].

Central deafness has been linked historically to bihemispheric involvement of the temporal lobe, with more recent findings suggesting that compromise of other cortical and subcortical structures and brain stem can also result in this disorder [50]. Individual with central deafness often presents with a rather dramatic auditory deficit. These patients often demonstrate inconsistent or no responses to sound [50].

Subarachnoid hemorrhage affecting both inferior colliculi can cause central deafness [50]. The bilateral involvement of auditory structures within the midbrain can result in this condition.

#### 2.3.2. Cerebral Vasospasm

A patient with SAH who developed sudden bilateral deafness [51] also experienced sudden onset of headache, nausea, and vomiting 9 days before referral to a hospital, where an old infarction was identified in the left temporal lobe before the occurrence of SAH. Hence, cortical deafness in this patient was caused by vasospasm contralateral to the previous infarction. Unilateral deafness that occurred after the rupture of a right vertebral artery dissecting aneurysm resulted from ischemia in the territory of the right internal auditory artery due to vasospasm [52].

Tabuchi et al. discovered a reversible cortical auditory dysfunction that was caused purely by bilateral cerebral vasospasm after aneurismal SAH [6]. Acute bilateral deafness that appeared 7 days after SAH onset was reversed by improving the cerebral vasospasm. This case suggests that transient ischemia involving the bilateral auditory cortices and auditory radiations can cause this unusual symptom [6]. A partly reversible central auditory dysfunction induced by cerebral vasospasm after SAH improved over a period of 6 months [7]. Whereas cortical deafness might have been associated with bilateral lesions of the temporal cortex, central auditory dysfunction was partly reversible in this patient after prominently unilateral right temporal lesions. The author considered the roles of interthalamic connections and less severe vasospasm of the left MCA that transiently impaired the left thalamocortical auditory pathways [7].

### 2.4. Cerebrovascular Malformation

#### 2.4.1. Arteriovenous Malformation (AVM)

Surgical excision of an AVM within the internal auditory canal (IAC) that caused sensorineural hearing loss did not affect complete deafness in one patient [53]. A cerebellar AVM was totally extirpated 122 days after initial symptoms resembling those of ear disease appeared, including unilateral facial palsy, hearing impairment, and tinnitus [54]. Sudden deafness has also arisen as a manifestation of a right temporoparietooccipital AVM, the rupture of which caused SAH [55]. An AVM can be life threatening if overlooked and thus it should be considered when hearing is disturbed.

#### 2.4.2. Dural Arteriovenous Fistula (DAVF)

Dural arteriovenous fistulae usually between the external carotid artery and dural venous structures are a rare entity accounting for 10%–15% of all cranial AVMs. Spontaneous closure (regression) of a DAVF in the middle ear has been associated with acute hearing loss [56]. Intraosseous DAVF of the skull base, possibly as a consequence of compression of the cochlear nerve or vasculature by a draining vein or nidus of the DAVF, has been associated with hearing loss [57].

#### 2.4.3. Cavernous Angioma (Cavernoma)

Intrapetrosal cavernous angioma involving the facial nerve is a well-known ontological entity [58]; however, cavernous angiomas rarely occur in the IAC. These tumors originate from the capillary bed of the epineurium surrounding the nerve and can either compress or infiltrate the nerve. These lesions can cause severe and progressive sensorineural hearing loss, tinnitus, facial nerve palsy, or vertigo even when they are relatively small [59]. All the patients initially presented with progressive sensorineural hearing loss, which can be caused by cavernous angiomas of the IAC [53, 59–62]. Cavernous angioma within the IAC can be attached to the facial, acoustic, or intermediate nerve. They are very rare and only 40 cases have been histologically proven and described in the literature [62]. A diagnosis must be based on the patient's symptoms together with CT and MR imaging features. Surgery is the treatment of choice and surgical approaches vary, depending on the size of the lesion, its location, and the severity and duration of preoperative hearing loss [59]. Such angiomas must be surgically extirpated while avoiding complications arising from bleeding into surrounding structures [62]. A prompt surgical treatment in acutely symptomatic patients provides a chance of complete regression of the clinical symptoms.

#### 2.4.4. Venous Angioma (Developmental Venous Anomaly)

A venous angioma arising within the IAC and expanding to the brainstem has caused a unilateral profound sensorineural hearing loss [63]. Hearing loss in this outpatient was incidentally discovered in the right ear. T2-weighted MRI revealed hypointense large tubular structures of the IAC that extended to the contralateral side of the brainstem across the right cerebellopontine angle area and multiple branching structures in the cerebellar hemispheres with intense contrast enhancement were consistent with venous angioma. Observation of the lesion was recommended until other intracranial complications developed [63]. Venous angiomas are low-flow, low-resistance lesions that are less likely to hemorrhage, the estimated overall risk of which is 0.22% per year [64]. The morbidity and mortality rates of surgical or radiosurgical removal or obliteration of venous angiomas to eliminate risk for hemorrhage are significantly higher compared with the natural history. Thus, observation is the recommended primary mode of therapy [64].

#### 2.4.5. Capillary Telangiectasia

Capillary telangiectasia is often found incidentally on MRI or at autopsy and may be associated with minor neurologic symptoms, but there has been little evidence about whether such lesions are responsible for these symptoms [65]. Sensorineural hearing loss and tinnitus abruptly developed in a patient with capillary telangiectasia of the pons that might have affected the auditory and vestibular central pathways in the right midpons [65]. Capillary telangiectasias are usually <2 cm in diameter, and they are most frequently located in the pons [66]. These lesions show slight contrast enhancement and an obvious gradient-echo signal loss on MR images, except when a draining vein is present. Capillary telangiectasias have often been incidentally discovered and treated conservatively [67], but they have also been associated with hemorrhage and progressive and aggressive neurological deterioration [68, 69].

Sudden deafness defined as a sudden loss of hearing due to unknown causes is often treated with vasodilators and drugs to improve blood flow. However, cerebrovascular malformations must be ruled out by MRI because their presence would contraindicate the administration of vasodilators and drugs to improve blood flow.

### 2.5. Moyamoya Disease (MMD)

Cortical deafness due to bilateral temporal subcortical hemorrhages associated with moyamoya disease (MMD) gradually improved and completely disappeared within two months [70]. Damage to the bilateral auditory radiation might have played a role in the cortical deafness arising in this patient.

Diffuse ischemic damage to the subcortical white matter including areas of the temporal lobes as well as multiple and focal cortical infarctions in both cerebral hemispheres determined by MRI in a 3-year-old boy with MMD and central deafness suggested thalamocortical dysfunction and disconnection between higher brainstem and cortical auditory areas [71]. Sudden unilateral hearing loss that was possibly caused by vascular occlusion resulting from thrombotic narrowing or blockage by plaque presented as the first symptom of MMD [72]. Therapy with high-dose steroids and a vasodilator resulted in only mild improvement [72].

Moyamoya disease is principally a bilateral progressive cerebrovascular disorder that can plausibly cause cortical and/or central deafness, either via ischemic or hemorrhagic lesions. The possibility of MMD should be considered especially in children, young adults, and East Asians with sudden hearing loss.

### 2.6. Superficial Siderosis (SS)

Superficial siderosis is associated with sensorineural hearing loss although it is generally considered to be rare and presumably underdiagnosed [73, 74]. SS of the central nervous system results from hemosiderin deposition in the subpial layers of the brain and spinal cord due to recurrent and persistent bleeding into the subarachnoid space [75]. Thus, SS might be associated with CVD. The source of bleeding remains unknown in about one-third of patients [76]. Sensorineural deafness (95%), cerebellar ataxia (88%), and pyramidal signs (76%) characterize SS [75].

Cerebellar Bergmann cells, microglia, and superficial astrocytes take up subarachnoid blood and then heme oxygenase intrathecally breaks down heme to free iron, which is cytotoxic as it produces reactive oxygen species. Both iron and heme products cause neurodegenerative injury [77]. Damage to the acoustic nerve can be prominent due to the nerve having a long course through the CSF within the pontine cistern and its investment in numerous microglial cells that become hemosiderin-laden [78].

The diagnostic modality of choice for SS is MRI [79], which can show characteristic marginal T2 hypointensity around the brain stem, cerebellum, and spinal cord. Vascular malformations associated with SS include cerebral AVM, spinal AVM, spinal AVF, cerebral aneurysms, and cavernous malformations [80–84]. However, bleeding sources might remain undetectable regardless of extensive neuroimaging. A 1995 survey of world literature revealed that the underlying cause of SS was identified in 34 of 63 patients [75].

Treatment options for SS have not been established. Surgical exclusion of a bleeding source is one therapeutic approach to stop SS progression, assuming that the bleeding source is identified. With respect to hydrocephalus, a CSF shunt is indicated when consciousness is disturbed [75]. Proposed medical treatments include iron chelators, desferrioxamine, trientine, and steroid [85, 86]. Deferiprone, which is a lipid-soluble iron chelator that can penetrate the blood-brain barrier, can improve the clinical symptoms and deposition of hemosiderin [87, 88] and thus provides hope for treating SS [89].

SS has been associated with cerebral amyloid angiopathy (CAA), which is an important cause of intracerebral hemorrhage in elderly patients [90, 91].

Compared to the well-described classical type of SS, which mainly affects brain stem and posterior fossa, SS in CAA showed a preference for the cerebral convexity and only exceptionally occurred in the infratentorial compartment [91]. SS might be recognized within the spectrum of CAA [92].

### 2.7. Auditory Function during the Chronic Stage of CVD

Risk of hearing loss is increased among elderly patients with stroke compared with the general population [93]. Stroke might result in disordered auditory processing [94] and one report describes auditory neglect in patients after cerebral stroke [95]. A substantial proportion of patients develop extreme auditory functional limitations not limited to speech sounds after stroke of the auditory brain [96]. Auditory function should also be evaluated during the chronic stage of CVD and the real incidence of auditory dysfunction in CVD should be elucidated.

## 3. Conclusion

Auditory dysfunction is a common clinical symptom that can profoundly affect the quality of life of affected individuals. As the aging population increases, more patients will present with CVD accompanied by auditory disturbances. Henceforth, CVD will probably become an important contributor to the differential diagnosis of auditory dysfunction. General physicians need to understand this likelihood.
